# What happens after drought ends: synthesizing terms and definitions

**DOI:** 10.1111/nph.18137

**Published:** 2022-04-30

**Authors:** Leena Vilonen, Maggie Ross, Melinda D. Smith

**Affiliations:** ^1^ Department of Biology Colorado State University Fort Collins CO 80521 USA; ^2^ Graduate Degree Program in Ecology Colorado State University Fort Collins CO 80521 USA

**Keywords:** drought, legacy effects, post‐drought period, post‐drought terms, recovery, resilience

## Abstract

Drought is intensifying globally with climate change, creating an urgency to understand ecosystem response to drought both during and after these events end to limit loss of ecosystem functioning. The literature is replete with studies of how ecosystems respond during drought, yet there are far fewer studies focused on ecosystem dynamics after drought ends. Furthermore, while the terms used to describe drought can be variable and inconsistent, so can those that describe ecosystem responses following drought. With this review, we sought to evaluate and create clear definitions of the terms that ecologists use to describe post‐drought responses. We found that legacy effects, resilience and recovery were used most commonly with respect to post‐drought ecosystem responses, but the definitions used to describe these terms were variable. Based on our review of the literature, we propose a framework for generalizing ecosystem responses after drought ends, which we refer to as ‘the post‐drought period’. We suggest that future papers need to clearly describe characteristics of the imposed drought, and we encourage authors to use the term post‐drought period as a general term that encompasses responses after drought ends and use other terms as more specific descriptors of responses during the post‐drought period.

## Introduction

Climate models predict an intensification of the hydrological cycle (Dai, 2011; IPCC, 2014; Asadieh & Krakauer, 2015). Increases in greenhouse gases are probably responsible for this intensification (IPCC, 2014), and if nothing is done to mitigate the rise in global temperatures (below the 1.5°C benchmark), drought will become more frequent, widespread, severe and long‐lasting over time (Cook *et al*., 2015; Lehner *et al*., 2017). This predicted intensification of drought has the potential to significantly impact future ecosystems, if past droughts are any indication of the future response (Cook *et al*., 2015). Indeed, extreme drought has been estimated to cause annual losses of about 1% of Earth’s terrestrial ecosystem function and reduce carbon (C) uptake by 0.14 PgC yr^−1^ globally (Du *et al*., 2018). With climate‐change‐driven intensification of drought, reductions in C uptake and more permanent losses in ecosystem function are expected to be magnified over time. As such, there is a pressing scientific need to understand how ecosystems respond to drought to better mitigate potential negative effects. Additionally, these effects may persist after drought has ended and affect ecosystem responses to future drought events (Schwalm *et al*., 2017). Thus, understanding the potential lasting effects of drought will be vital in developing Earth system models that can predict the true impact of drought both during and after these events.

The existing literature on ecosystem responses during drought is synthesized in both reviews (e.g. Niu *et al*., 2014; Felton & Smith, 2017) and metaanalyses (e.g. He & Dijkstra, 2014; Sun *et al*., 2020; Castagneri *et al*., 2021), which provide a cohesive narrative of the impacts of drought on a myriad of ecosystem processes as these events unfold. Although there is still uncertainty in drought responses, this body of work allows us to begin to generalize ecosystem responses during drought. For example, there is strong evidence from metaanalyses that the mean effect of drought on aboveground productivity is negative (Wu *et al*., 2011; Gazol *et al*., 2020; Sun *et al*., 2020). Yet, our knowledge of drought responses is incomplete without understanding whether responses that occur during drought persist after drought and for how long they persist. The current literature is largely inconsistent (Stuart‐Haëntjens *et al*., 2018) and limited in how ecosystems respond after drought, and whether and how long drought effects persist after these events end. Models often assume that ecosystems recover completely after drought ends, when in reality full recovery may take a few years (Anderegg *et al*., 2015) or may extend over decades (Weaver, 1944). The directionality of ecosystem response post‐drought is often mixed, with some research showing positive effects (e.g. increase in ecosystem functioning) post‐drought (Griffin‐Nolan *et al*., 2018; De Long *et al*., 2019), with others finding negative or neutral effects (Rousk *et al*., 2013, 2013; Hofer *et al*., 2016, 2016; Kreyling *et al*., 2017). Sala *et al*. (2012) found that legacies of dry years or low precipitation had negative implications for the next year’s growth, indicating that growth is inhibited after drought. This inconsistency in the directionality of responses could be driven by a myriad of factors, making it difficult to synthesize the literature in a cohesive way.

A synthetic understanding of ecosystem response after drought ends is further compounded by the various and inconsistent terms that researchers use to describe this period. As we describe in detail below, our review of the literature found these terms include legacy effects, lag effects, resilience, recovery, rewetting, drought memory and compound drought/double‐stressed. We contend that before we are able to synthesize the literature and move forward with research in this area, we need to unify these terms and definitions. This paper aims to summarize how researchers use and define these terms, discuss potential biases in this literature, and make recommendations on how we can best combine these terms in a unifying framework to allow for a generalized understanding of how ecosystems respond after drought events. To accomplish these objectives, we conducted a literature review focused on papers that examined above‐ and belowground terrestrial ecosystem responses after drought events have ended.

## Literature review

We conducted a literature review (Web of Science) to assess how researchers define and use terms related to ecosystem responses after drought ends. Based on an initial review of the literature, we identified the following terms for our search: ‘drought AND legacy effect*’; ‘drought AND memory effect*’; ‘drought AND lag effect*’; ‘drought resilience’; ‘drought recovery’; and ‘compound drought OR compounded drought’. We did not filter by year or subject area so as to not miss any possible papers. This February 2021 search yielded 1415 results, of which we deemed 94 papers relevant (Appendix A1). A large majority of the papers from this search were excluded because they did not impose drought, mentioned drought only in passing or were not conducted during the post‐drought period. Furthermore, many of the papers were experiments that mentioned drought in their abstracts but did not actually study drought while others were not ecological papers and were therefore excluded. From each relevant paper, we extracted information regarding ecosystem type; whether above‐ or belowground measurements were taken; whether the experiments were glasshouse, lab, field or remote sensing experiments; the reduction in precipitation that occurred; the length of drought; the time after drought that the measurement(s) was/were taken; whether there were one or two droughts; what term they used; how they defined that term; whether the effect was positive, negative or neutral; whether the mechanisms for the effect were abiotic or biotic; and the mechanisms cited for the effects observed (Appendix A1). A few of the 94 papers did not contain all of this information, so some categories had fewer than 94 entries.

## Terms, definitions and context of terms

### Terms and definitions

**Fig. 1:**
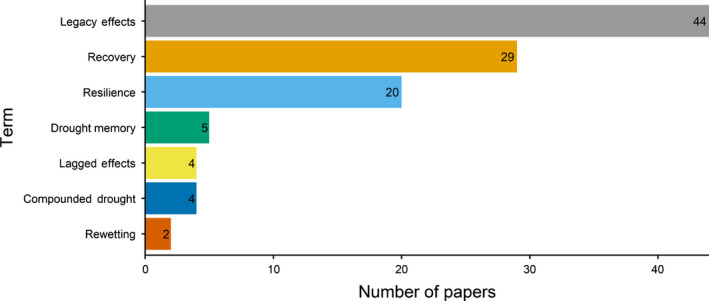
Summary of the terms used in the 94 papers reviewed and the number of papers that used each term.

The 94 papers included in our review most commonly used the term legacy effect(s) (41%), followed by recovery (27%), resilience (19%), lag effect (4%), compound(ed) drought (4%), memory (4%) and rewetting (2%) (Fig. 1). Legacy effects, recovery, resilience, lag effect and rewetting all describe the period after typically one drought and the response seen during this period. The term legacy effect was the most common term used probably because it is a simple way to say that effects were present after the end of drought. Recovery and resilience also describe this post‐drought period, whereas compounded drought and drought memory generally describe the response during a second drought. Rewetting can be considered different from the other terms, because it implies a larger than average level of precipitation, which the other terms do not imply.

After identifying the frequency in which terms were used, we extracted definitions authors used to describe the term or terms used in their papers. Based on the definitions provided by the authors, we generated a list of definitions commonly used for each term (Table 1). Generally, the most common definition used was ‘the effects of drought after drought has subsided’ (Table 1). The next most common definition used was ‘the ability to recover’. These terms were mostly associated with legacy effects and recovery/resilience, respectively. Terms were also defined with respect to the capacity to recover after drought, reduction in function, antecedent conditions, compound effects or departure from typical growth. Only a small percentage (8%) of papers did not include explicit definitions of the terms used.

**Table 1:** Summary of the definitions of each of the terms assessed in this review.

Term	Definitions	Reference
Legacy effect	Effects of drought after drought has subsided	Griffin‐Nolan *et al*. (2018)
	Indirect rather than direct effects of drought	Hicks *et al*. (2018)
	Lasting physiological changes	Kannenberg *et al*. (2019)
	How community responds after drought to rewetting	de Nijs *et al*. (2019)
	Lag or incompleteness in recovery	Huang *et al*. (2018)
Recovery	Growth reaction following drought period	Gazol *et al*. (2017)
	Post‐drought conditions/drought conditions	Vitali *et al*. (2017)
	Well‐watered conditions after drought	Panke‐Buisse *et al*. (2020)
	Time it takes to recover after drought	He *et al*. (2018)
Resilience	Capacity to recover to pre‐disturbed conditions	Dang *et al*. (2019)
	Ability to recover from drought events	Elsalahy *et al*. (2020)
	Post drought conditions/ pre‐drought conditions	Vitali *et al*. (2017)
	Post‐drought recovery rate	Li *et al*. (2020)
Lag effect	Positive correlations the following year after drought	Zhao *et al*. (2018)
Drought memory	Memory that helps respond to future disturbance	Leufen *et al*. (2016)
	Persistent effects of antecedent precipitation on productivity	Liu *et al*. (2018)
Rewetting	Wet period after drought	Van Sundert *et al*. (2020)
Compounded drought	Effect of old perturbation to new perturbation	Peltier & Ogle (2019)
	Effects of heatwave and drought at one time	El‐Madany *et al*. (2020)

The different definitions for each term are provided with references for papers in which the terms were used and defined. Our goal was to include the general definitions found across the papers and cite the most relevant papers.

### Context of terms

**Fig. B1:**
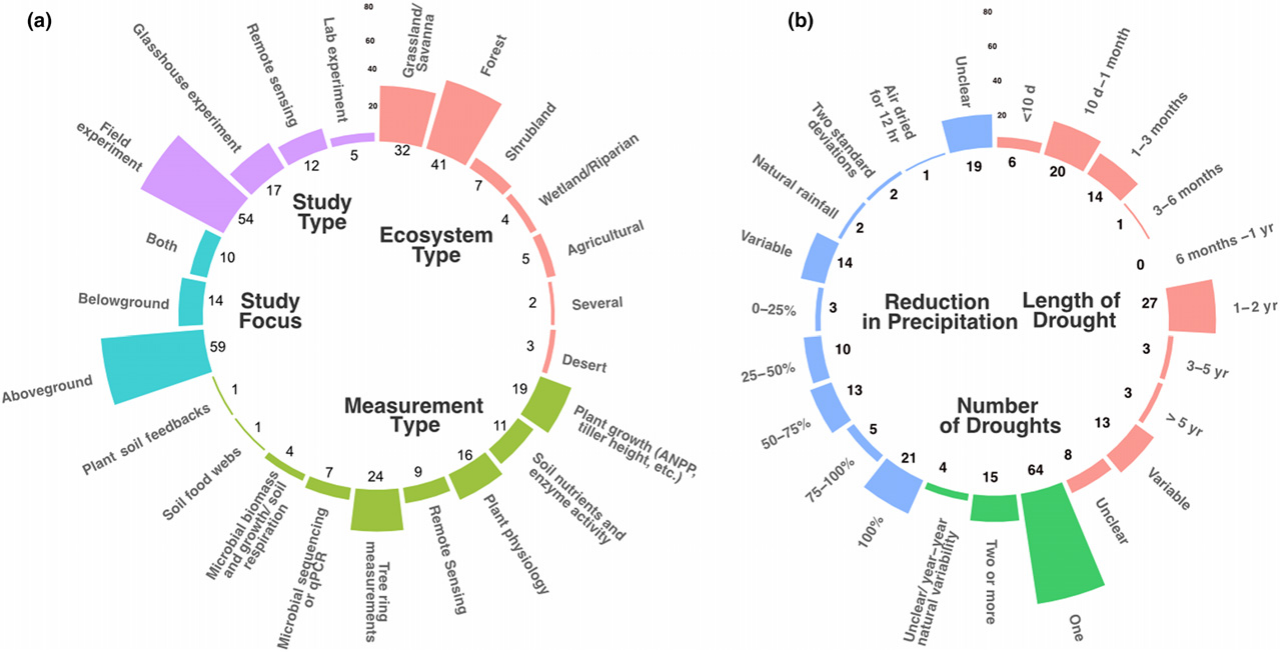
Key descriptors of (a) study characteristics and (b) drought characteristics the 94 studies included in the literature review. Study characteristics included: ecosystem type (orange bars), study type (purple bars), study focus (aqua bars) and measurement type (green bars). Drought characteristics included: reduction in precipitation (blue bars), number of droughts (green bars) and length of drought (orange bars). Numbers at the base of each bar indicate the number of studies that fell into a category of each characteristic.

With our literature review, we extracted a suite of study attributes to determine if there were specific contexts in which terms were used (Box 1). These study attributes included: ecosystem type, study type, measurement type, and types and direction of responses measured, as well as the time after drought measurements were made (Fig. B1a). In addition, we also examined key characteristics of the drought itself (Fig. B1b). We found that several study attributes and drought characteristics stood out for differentiating the context in which terms are used.

Box 1 **Attributes of the studies and characteristics of drought**

The 94 studies included in our literature review varied in their study attributes and characteristics of drought. We were able to extract several study attributes including: ecosystem type; whether above‐ or belowground measurements were taken; whether the experiments were glasshouse, lab, field or remote sensing experiments; and what measurements were taken. For studies that fit into more than one category (e.g. plant growth and nutrient analysis for measurement type), we counted the paper separately for each category they fell into. The drought characteristics examined were the reduction in precipitation that occurred; the length of drought; and whether there were one or two droughts. We found that studies were mostly from grassland and forest ecosystems, were mostly tree ring and plant growth measurements, consisted of mostly aboveground measurements and were mostly field experiments (Fig. B1a). We also found that most studies imposed one drought and varied considerably in length of drought and reduction in precipitation imposed, although most studies imposed drought under 1 yr and had mostly unclear (not mentioned in the paper) reductions in precipitation (Fig. B1b).

**Fig. 2:**
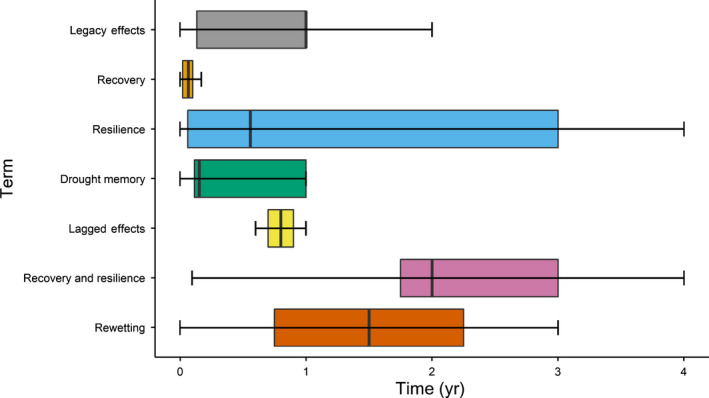
Box plots with median bars (outliers not shown) showing the average time after drought that each study measured responses with respect to the terms used. The boxes represent the interquartile range that contain the 25^th^ percentile and 75^th^ percentile range of the data. The whiskers represent 1.5 times the interquartile range for the minimum and maximum whisker. The category ‘recovery and resilience’ includes papers that used both the terms recovery and resilience, while the categories ‘recovery’ and ‘resilience’ include papers that only used the terms individually.

One such study attribute was the average time after drought that studies measured post‐drought responses (Fig. 2). When the terms recovery and resilience were used simultaneously, researchers measured the post‐drought responses on average 2 yr after the drought ended, which was longer than when other studies measured their responses after drought. Resilience and rewetting both individually on average measured responses 1.5 yr after the drought ended. It is logical that papers that measured recovery and resilience would measure the effects post‐drought at a longer time scale, since the papers claim to see recovery after some sort of time scale, which leads to the papers calling the system resilient. It is also possible that studies focused on particular vegetation types, such as forests, favor the terms resilience and rewetting which are often measured on a longer time scale. Investigators measured post‐drought responses *c*. 1 yr after the event ended when using the terms recovery, legacy effects and lagged effects. If a legacy effect or lagged effect is still occurring, this is probably closer in duration to when the drought ended. Lastly, drought memory studied the effects after drought on average after 0.5 yr. Although the terms differed in on how long on average investigators measured the effects post‐drought, the time frame in which post‐drought responses/effects were measured averaged around 1 yr after drought (range 0–20 yr), which could be too short‐term, since most papers observed lingering effects from the drought.

**Fig. B2:**
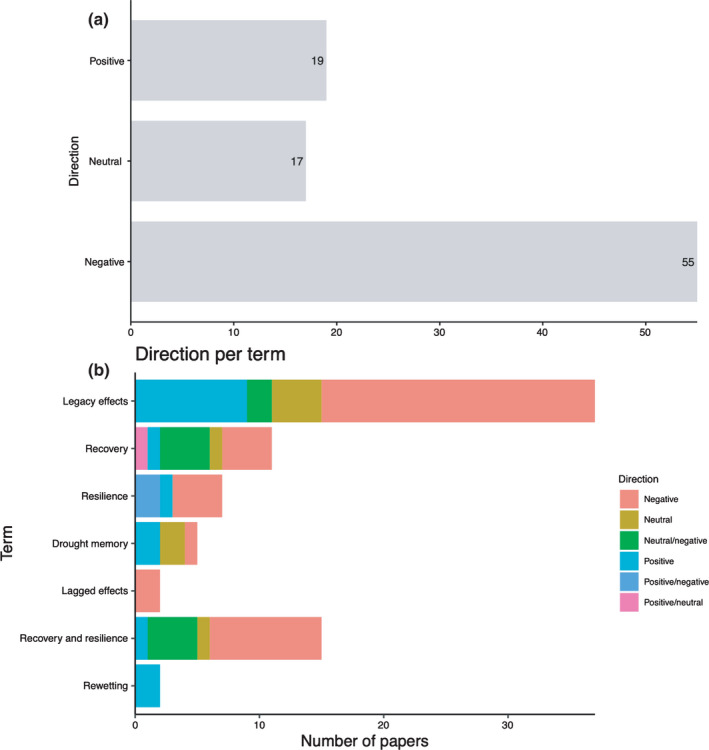
(a) The number of responses that were positive, negative or neutral in the 94 papers reviewed. (b) The number of papers reporting a direction of response (positive, negative, neutral or combined) for each term.

Another study attribute that provides important context for the post‐drought terms usage was the direction (positive, negative or neutral) of post‐drought responses measured (Box 2). We found that the 94 papers reviewed disproportionally reported negative effects of drought (Fig. B2a). While it is possible that negative effects are more common than neutral or positive effects, it is not possible to distinguish this from a publication bias. This bias could skew future metaanalyses or syntheses toward more negative results than the true value of post‐drought responses. Therefore, it is important to encourage the publishing of neutral (otherwise known as negative) results (Mlinarić *et al*., 2017). In addition to an overall bias in direction reported, we also found that the direction of response differed by term used by a study (Fig. B2b). Papers using the terms legacy effects and lagged effects reported mostly negative responses after drought. By contrast, drought memory reported a variety of different responses (neutral and negative responses), even though one might expect only positive responses being reported. Recovery and recovery/resilience papers had more neutral and positive responses than negative responses. Finally, papers using resilience reported an equal number of positive and negative responses, while rewetting papers reported only positive responses.

Box 2 **Direction of post‐drought responses published**

We identified papers as positive if the response after drought was positive (e.g. increased plant growth or increases in soil nutrients), negative if the response was negative (e.g. losses in plant growth or loss of soil nutrients) or neutral if there was no significant response found (e.g. plant growth was the same as the control or predisturbance). We counted the number of studies that by our definition had a positive, negative or neutral response. When papers had both neutral and negative effects or neutral and positive effects, we counted each effect as a separate entry. We conducted a chi‐square test for the number of papers that had positive, negative or neutral responses post‐drought to determine if the difference observed was by chance. Overall, 19 studies had positive effects, 55 had negative effects and 17 had neutral (or no) effects. To assess if there was bias in the publications, we used R statistical software and the base R function ‘chitest’ to obtain our *P‐*value (R Core Team, 2013). A significant *P*‐value (*P*< 0.05) would indicate that our results were not due to chance and there is a bias involved. The test produced a significant *P*‐value (< 0.001), which supports that reported results were biased toward those that are negative. We checked if this bias applies to just legacy effects papers, which was the category with the largest number of papers. There were nine studies with positive effects, 26 with negative effects and six with neutral effects. We found a significant *P*‐value in this test as well (*P* = 0.002), indicating that these differences were not due to chance.

## A synthesis of post‐drought terms and definitions

**Fig. 3:**
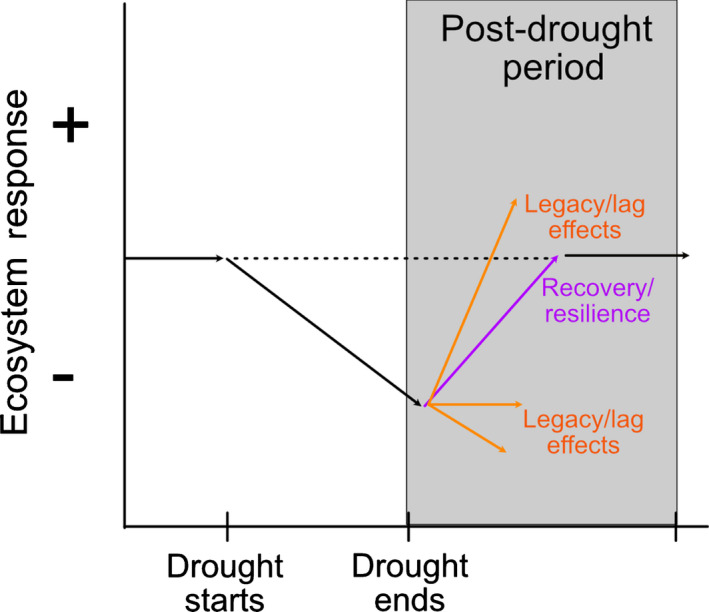
Framework for describing ecosystem responses after drought ends. We refer to this as the ‘post‐drought period’. Within this post‐drought period, we propose that the nature of the ecosystem response can be described by a set of terms. The start of the drought refers to either one drought or any subsequent droughts. We propose that the same terms should be used during the post‐drought period regardless of how many drought periods have occurred. Legacy (which includes lag) effects describe either positive or negative effects observed during the post‐drought period. Recovery and resilience are terms that describe neutral effects seen after a certain amount of time following drought.

Our review illuminated the variety of terms used to describe ecosystem responses after drought ends and the variability in definitions of these terms and the contexts in which they are used. To provide a cohesive framework, we propose using the term ‘post‐drought period’ to describe ecosystem responses that are observed after a drought ends (Fig. 3) and using the terms highlighted in this paper to further describe the nature of the ecosystem responses observed in the post‐drought period.

The first most commonly used term in the papers we reviewed was legacy effects. Legacy effects and lag effects have similar definitions and thus can be used interchangeably. However, the term legacy effect(s) is more commonly used over lag effects, and we suggest it is the more appropriate term to use for describing responses during the post‐drought period. Furthermore, we propose that legacy effects be used to describe responses observed in the post‐drought period, whether they are positive or negative. These effects can last for an undefined period of time or could be indefinite. Sometimes the changes may be irreversible, indicating a state change, which would also be an example of a legacy effect. The term legacy effect(s) is most appropriate for describing the directionality of effects but would not be appropriate for describing a neutral effect. Legacy effects would also not be appropriate for describing the post‐drought period generally, since it is a descriptor of what occurred not a temporal description of the period after drought. As we observed across the papers used in this study, not all responses were positive or negative. Indeed, 19% of the papers had a neutral response, which would make legacy effects inappropriate for describing such responses, since our framework argues that legacy effects must have a directional response (Fig. 3). Additionally, there actually may be more instances of post‐drought neutral responses if potential publication biases are eliminated as discussed above. Lastly, legacy effects have also been described as the effect of the past year’s precipitation (Sala *et al*., 2012) or as historical conditions (Bunting *et al*., 2017), and we suggest that these be called antecedent conditions to avoid confusion with our definition of legacy effects.

The second and third most commonly used terms were recovery and resilience. Both terms are different from legacy effects because they describe a trend towards pre‐drought conditions, but they are generally quantified in different ways (e.g. mathematical equations in ecophysiology; Table 1). If there is a negative or positive response observed post‐drought, but the response returns to pre‐drought levels, then by definition the system has recovered. Additionally, if a system recovers quickly or is not largely affected post‐drought or during the drought event (i.e. neutral response), then we propose the system be called resilient to drought. Some systems may never recover after drought or experience a state change, which would make recovery inappropriate and misleading to use for describing the post‐drought period. Given that systems may vary in how long it takes to return to pre‐drought conditions, recovery is highly dependent on the time scales considered. Thus, recovery and resilience are appropriate terms when describing the directionality or speed of return to pre‐drought conditions (otherwise known as a neutral response), but we contend these terms are not appropriate for generally describing the post‐drought period.

The terms compounded drought and drought memory both describe the time point when a second drought occurs after the first drought ends. The two terms differ in their implication of the direction of the response. Drought memory implies a positive response to a second drought. The term memory implies that there is a ‘remembered’ effect from the first drought that assists with the response to a second drought. Drought memory would in many cases be inappropriate for describing a response but would be best used to potentially justify or explain positive responses (legacy effects) if they are observed. Compounded drought depicts the sequential occurrence of drought events within a certain period and is not related to the characteristics of ecosystem responses. We suggest calling the period after a compounded drought the post‐drought period and using the terms legacy effects, resilience and resistance in the same way as after a single drought event to describe the nature of the response. It is important to note that compounded drought has also been defined as another perturbation such as a heat wave occurring at the same time as drought (Matusick *et al*., 2018; Zcheischler *et al*., 2018; El‐Madany *et al*., 2020). Thus, we suggest using sequential drought to describe two or more drought events and use compounded drought to describe the co‐occurrence of drought with heatwaves or other perturbations.

A key finding from our review is that the length of time of the post‐drought period captured by the studies was often inconsistent or even undefined. We were able to assess this by looking across the terms used and the average amount of time that the paper measured responses post‐drought (Fig. 2). As discussed earlier, we found that the average time that these studies measured responses post‐drought was relatively short. Although some studies measured drought effects up to 20 yr post‐drought, most studies measured the effects of drought on above‐ and belowground ecosystem responses for <1 yr after the drought occurred. This is a short time frame and could also explain the bias we discussed towards negative results. Perhaps more papers would have observed a neutral effect had the responses been measured over a longer time scale or perhaps negative effects may have persisted longer than studies currently measure post‐drought responses. This highlights the need for post‐drought studies to measure responses for a longer time scale, particularly if they are interested in determining whether the system recovers or is resilient. Furthermore, many papers used the term recovery, yet they saw directional responses over the study period (Fig. 2). Very few of the recovery and resilience papers saw full recovery and classified their effects as negative, since most still had negative responses. Using recovery or resilience for systems that have been unable to recover or have not yet recovered is misleading. These are instances when the term legacy effects would be more appropriate for describing the response in the post‐drought period. Timescales will be vital in future post‐drought research with a strong preference for longer term experiments along with defining the characteristics of drought clearly.

## Mechanisms underlying post‐drought responses

**Fig. B3:**
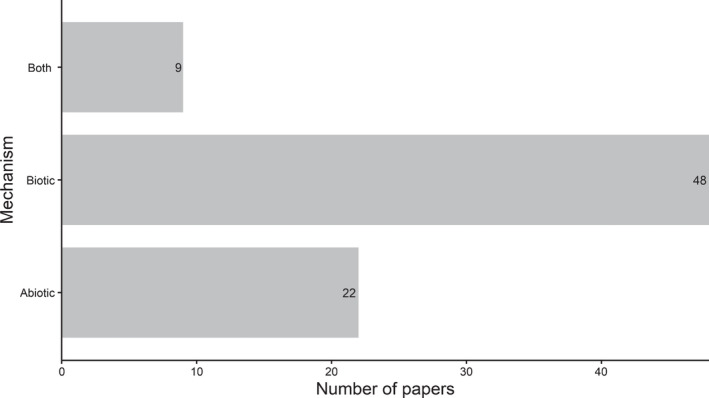
The number of papers that cited biotic, abiotic, or both abiotic and biotic mechanisms for the responses that papers found post‐drought.

The papers reviewed attributed various mechanisms to explain positive, negative or neutral post‐drought responses. Papers mostly cited biotic reasons (60%) as the only mechanism responsible for the effect in the study (Fig. B3). Additionally, most papers cited a physiological reason as the mechanism for the response observed (Supporting Information Table S1). Biotic, particularly physiological, mechanisms imply that the responses were plant‐driven, which is highly possible, although belowground processes could also contribute to the aboveground responses observed. Some papers (18%) cited belowground reasons as the mechanisms such as changes in nutrient concentrations, elevated nitrogen levels, microbial community‐mediated, less active microbial community or microbial turnover of plant carbon (Table S1). Other papers most commonly cited water reserves or precipitation‐based reasons as the mechanisms for the responses measured. Various mechanisms were used to explain responses observed in the papers, but it is unclear which mechanisms are most important and drive these effects. The biggest problem is that studies typically only cited one or a few mechanisms, which is unlikely to be the case in reality. This field of research will need to be driven forward by studying general mechanisms, focusing on mechanisms that link below‐ and aboveground processes and responses. It is highly unlikely that the mechanisms (e.g. physiological or nutrient‐mediated) in the post‐drought period driving the responses, whether it be recovery or a state‐change, will be driven by only a single factor, as several factors have been shown to improve recovery post‐drought (Xu *et al*., 2013, 2013; Jiao *et al*., 2021). This field of research would benefit from studies that holistically examine the mechanisms driving the responses seen during the post‐drought period (Box 3).

Box 3 **Mechanisms underlying post‐drought ecosystem responses**

Mechanisms were grouped into categories (below) and then the numbers of papers that fit into these categories were counted. Mechanisms were determined by each paper’s reasoning for the response post‐drought typically highlighted in the discussion section. These mechanisms were then grouped and compiled as in Supporting Information Table S1. We then split these into biotic and abiotic mechanisms as in Table S1 and counted the number of papers that cited biotic only, abiotic only, or both abiotic and biotic mechanisms that led to the response seen post‐drought (Fig. B3). Papers mostly sited biotic responses and changes in physiology as the reasons for the response seen.

## Knowledge gaps

Overall, our review suggests that we have limited understanding of the period after drought due to a dearth of studies and an undue emphasis on aboveground ecosystem responses, with potential publication biases making it difficult to parse out what happens after drought. Indeed, 71% of the studies examined aboveground ecosystem responses alone, which leaves a large gap in the knowledge of belowground responses after drought (Fig. B1a). There is a pressing need to understand belowground responses post‐drought, since the belowground realm serves important functions such as nutrient cycling, decomposition and carbon sequestration. Yet, our review suggests that belowground ecosystem responses are generally understudied, warranting further research.

Furthermore, as a body of research in ecology matures, metaanalyses become an important way in which results from numerous studies can be synthesized to find generality (Gerstner *et al*., 2017), since ecological responses are often variable and occur at a large scale. Metaanalyses of responses after drought will be important for providing general understanding of post‐drought responses, which is critical in mitigating potential negative impacts of drought. At this point in time, we are approaching enough papers for a robust metaanalysis of aboveground responses in the post‐drought period, although most papers focus on plant growth and tree ring measurements (Fig. B1a). A metaanalysis becomes even more challenging for belowground responses. The only statistical analysis we were able to conduct in our review was a chi‐square test to test for publication bias, because the number of papers were insufficient for any further analysis such as effect size of different ecosystem responses. This review has highlighted the absence of post‐drought research, particularly that focuses on belowground responses. Furthermore, our review revealed the variability in the ways in which drought is imposed and the resultant responses that were observed (Fig. B1b). Adding to the post‐drought research literature and using standardized approaches to imposing drought will allow for improved metaanalysis and synthesis in the future and increased understanding of post‐drought ecosystem responses.

Research on the post‐drought period will also be difficult to synthesize because researchers define drought inconsistently and the characteristics of drought are not clearly described (Fig. B1b; e.g. the timing, length and magnitude of drought). Variability between how studies conduct drought research is inevitable, but it is crucial for papers to explicitly describe the characteristics of their drought even it was a natural drought. One such characteristic was the time that the response was measured after the drought ended. In total, 11% of studies had variable timing of their measurements post‐drought, and in 5% of studies it was unclear at what time they measured effects post‐drought. Papers were even more inconsistent in describing the magnitude of the drought. In 21% of the studies, we were unable to determine the magnitude of reduction in drought. The length of the drought period also was generally not explicit, with 15% of studies having variable lengths of the drought period and 10% were unclear in their length of the drought period. It is clear from this review that a common definition of drought needs to be defined, as is discussed in Slette *et al*. (2019), and that papers need to clearly articulate the characteristics of their drought.

A second knowledge gap is the study of compounded droughts. Only 7.4% of papers used compound(ed) drought, double‐stressed or drought memory to describe the effect of a second drought after a previous drought has occurred in the system. This could be important for future understanding of a drying climate because subsequent drought events may occur before the system has recovered from a previous drought (Schwalm *et al*., 2017).

A third knowledge gap is the need for further research examining the mechanisms underlying post‐drought ecosystem responses. We found that the mechanisms proposed were variable, but primarily focused on biotic mechanisms related to plant ecophysiology. How plants may be responding below ground and affecting soil processes and vice versa were lacking as potential mechanisms. This is important because plant–soil feedbacks and specifically the potential for decoupling, in which above and below ground have different responses to drought and their interaction is changed, is probably an important in affecting post‐drought responses (Bardgett *et al*., 2013, 2013). For example, if the soil microbial community changes after drought, but the plant community does not, functional decoupling could occur, because the interactions between the above‐ and belowground processes will change. It will not be enough to only study above‐ and belowground effects and mechanisms separately because studies on plants and soil must be combined to measure potential decoupling and the feedbacks between them (van der Putten *et al*., 2016).

## Conclusions

Our review highlights the need for consistency of the terms used to describe the post‐drought period and the knowledge gaps needed to advance research aimed at elucidating the effects of drought after these events end. Our review found that papers use a variety of terms to describe the period after drought, often do not fully describe drought characteristics, are short term in their study of post‐drought responses and have potential biases that may impede future synthesis. Our review aimed to bring together the terms used to describe post‐drought responses and proposed a common framework for these terms, which we refer to as the ‘post‐drought period’. Within this post‐drought period, we propose that terms often used to describe responses after drought ends be used as descriptors of the nature of post‐drought responses rather than a description of the period itself. We further propose that the term sequential drought be used to describe a drought event occurring after a previous drought event, but that the time after each drought event be called the post‐drought period and use consistent terminology for describing the nature of post‐drought responses. We hope that papers will use our framework to increase consistency among studies. We also propose determining whether publication bias exists based on the preponderance of negative ecosystem responses reported following drought; conducting more research on the mechanisms underlying post‐drought responses, plant–soil feedbacks, and the decoupling of above‐ and belowground processes; and better describing of the characteristics of the drought itself. These knowledge gaps must be remedied to provide a comprehensive and predictive understanding of ecosystem responses during the post‐drought period, which almost certainly represents a longer period of impacts than those occurring during drought events.

## Author contributions

Contributed to conception and design: LV, MDS. Contributed to acquisition of data: LV, MR. Contributed to analysis and interpretation of data: LV, MR, MDS. Drafted and/or revised the article: LV, MR, MDS. Approved the submitted version for publication: LV, MR, MDS.

## Supporting information


**Table S1** Summary of the mechanisms that papers cited as the reason for the response observed.
Please note: Wiley Blackwell are not responsible for the content or functionality of any Supporting Information supplied by the authors. Any queries (other than missing material) should be directed to the *New Phytologist* Central Office.

## Data Availability

All data are publicly available at GitHub: https://github.com/Leena312/postdroughtreview‐newphytologist.
