# Supplementary material for: What happens after drought ends: synthesizing terms and definitions
Source: New Phytol. 2022 Apr 30;235(2):420–31. doi: 10.1111/nph.18137 (PMC9322664; doi:10.1111/nph.18137)
Supplement: Supplementary file 1 — Table S1 Summary of the mechanisms that papers cited as the reason for the response observed. Please note: Wiley Blackwell are not responsible for the content or functionality of any Supporting Information supplied by the authors. Any queries (other than missing material) should be directed to the New Phytologist Central Office. [file NPH-235-420-s001.pdf]

New Phytologist **Supporting Information**

Article title: **What happens after drought ends: synthesizing terms and definitions**
Authors: Leena Vilonen, Maggie Ross, and Melinda D. Smith
Article acceptance date: 18 March 2022

The following Supporting Information is available for this article:

Table S1. Summary of the mechanisms that papers cited as the reason for the response observed.

Mechanisms	Number of papers
**Biotic**	
Changes in physiology	13
Foliage losses	5
Loss of trees/ evapotranspiration	1
Species trait shifts	1
Photoprotection	1
Reductions in photosynthesis	1
Root to shoot ratios	1
Plant community change	2
Seedling establishment	1
Microbial community mediated	4
Higher levels of microbial biomass	1
Less active microbial community	1
Microbial turnover of plant-derived C	1
Age/height of plant	4
Effect of neighboring trees	2
Root exudation	2
Root regeneration/growth	1
Stomatal and biochemical traits	1
Plant enzyme activity change	4
Prioritization of leaf flushing	1
Biomass allocations and carbohydrate metabolisms	1
Carbon reserves	5
Storage of carbohydrates	1
Loss of carbohydrates	2
**Abiotic**	
Water reserves left over from year before	5
Change in nutrient concentrations	5
Elevated nitrogen levels	3
Increased light availability	2
Precipitation related (water amount)	5
Unclear	5
